# The impact of COVID-19 pandemic on total treatment time of fixed appliances

**DOI:** 10.1186/s40510-022-00437-0

**Published:** 2022-09-06

**Authors:** Milena Santos, Guilherme de Araujo Almeida, David Normando

**Affiliations:** 1 Federal University of Pará, Street Augusto Correa , Belém, Pará 66075-110 Brazil; 2 Federal University of Uberlândia, Avenue Pará, Uberlândia, Minas Gerais 38401-136 Brazil

**Keywords:** COVID-19, Corrective orthodontics, Pandemics

## Abstract

**Background:**

Several aspects of the orthodontic routine seem to have been affected since the emergence of SARS-CoV-2. We aimed to evaluate the impact of the COVID-19 pandemic on the duration of fixed orthodontic treatment.

**Methods:**

This retrospective study evaluated consecutive cases of patients undergoing fixed orthodontic treatment that completed treatment before (*n* = 37) or during (*n* = 26) the COVID-19 pandemic. The impact of the pandemic on treatment time was adjusted for the patient’s initial age, sex, number of debonds/breakages, number of missing teeth, initial PAR (Peer Assessment Rating) index (T0) and operator (*n* = 2), through multiple linear regression. The impact generated by months of treatment conducted during the pandemic period was also examined. Seven poorly finished cases were previously excluded, including five finished during the pandemic.

**Results:**

Although the number of absences/missed appointments of patients treated during the pandemic was four months more than those treated in the previous period (*p* < 0.001), there was no significant effect of the pandemic on total orthodontic treatment time for both operators. There was also an effect of operator (*β* = 10.42, *p* < 0.001) and gender, which was lower in females (*β* = 4.77, *p* = 0.03), on treatment time (*R*^2^ = 0.27). The other variables showed no significant association (*p* > 0.05).

**Conclusion:**

The COVID-19 pandemic did not have a significant effect on total orthodontic treatment time, although a greater number of absences/missed appointments were observed.

## Introduction

In December 2019, a new respiratory illness caused by the SARS-CoV-2 virus was first detected in China. COVID-19 [1] was officially declared as a pandemic by the World Health Organization (WHO) on March 11, 2020 [2]. Some restrictions on dental offices have become necessary due to the high potential for transmission of COVID-19. Regarding orthodontics, most active orthodontic treatments were immediately suspended, and face-to-face consultations were restricted to urgencies and emergencies [3, 4]. For this reason, thousands of orthodontic patients in epidemic areas postponed their appointments, most of them performed monthly, due to the elective nature of the treatment [5]. Consequently, the reduction of activities in the offices, the cancellation of appointments and the reluctance of patients to schedule their appointments have a potential direct impact on the duration of orthodontic treatment.

The pandemic seems to have an impact on the anxiety of orthodontic patients [6–8], especially among women [7, 8], where the delay in completing the treatment seemed to be their biggest concern [7]. Previous studies [9–11] have shown that several factors, especially those related to patient cooperation, can significantly explain the variability in treatment time; among them are absences from appointments and appliance issues/breakages.

Previous reports have evaluated the impact of the pandemic on the quality of finished cases and orthodontic treatment time [12, 13] and have found no clinical differences between the rates of occlusal improvement of patients treated before or during the pandemic. Regarding treatment time, there is a study reporting no significant impact [12], and one an increase in the treatment time of patients treated during the pandemic [13]. The control of confounding variables that impact treatment time in these studies was not carried out.

Several aspects of orthodontic treatment may have been affected since the emergence of the new coronavirus, and it is important to understand, among other variables, the impact of the COVID-19 pandemic on duration of orthodontic treatment. While there was an inevitable distancing of patients from the office, teledentistry minimized the inherent effects [14]. Therefore, the objective of this study was to evaluate the impact of the COVID-19 pandemic on fixed orthodontic treatment time, corrected for potential confounding factors.

## Material and methods

### Ethical consideration

This study was submitted to the Ethics and Research Committee of the Health Sciences Center of the Federal University of Pará and approved under protocol number 51451621.5.0000.0018.

### Study design, participants and eligibility criteria

This is a retrospective observational study that followed STROBE [15] guidelines for writing. The records of patients treated consecutively by two operators with more than 20 years of experience in orthodontics were evaluated. Patients treated in private offices with permanent dentition at the beginning of treatment, treated with fixed orthodontic appliances (brackets Straight-Wire slot 0.022″ × 0.028″) and with complete documentation were included. Patients transferred from other clinics, surgical cases, patients with craniofacial syndromes and/or cleft lip and palate, patients with impacted teeth, cases considered poorly finished (PAR index > 5) and patients with > 13 missed appointments were excluded. Orthodontic treatments completed between January 2018 and February 2020 were grouped as before the official emergence of the COVID-19 pandemic, while treatments completed between March 2020 and April 2022 were considered as treatments performed during the pandemic.

### Analyzed variables, data sources, measurements and sample size calculation.

The following data were collected from clinical and orthodontic records: patient’s initial age, sex, year and month in which treatment was completed (before or during the pandemic), total treatment time in months, number of absences/missed appointments; number of debonds/breakages of orthodontic accessories, missing teeth and the time in months that the treatment was carried out during the pandemic period. The beginning of treatment was defined as the moment the brackets were bonded and the end of the treatment when the appliance was removed. Absences/missed appointments were considered when the patient did not return in each month or when the orthodontist made it clear that the patient had been absent. The number of debonds/breakages was identified as the number of bracket repositioning and band re-cementation due to fracture or bond failure. The time in months in which the treatment was conducted during the pandemic was considered from March 2020, when COVID-19 was identified in the cities where the two orthodontists attended, until the time that the treatment was finished.

The following data were collected from the models: number of missing teeth, confirmed by panoramic radiography and PAR [16] index. All plaster models were scanned (TRIOS® Pod 3Shape, Copenhagen, Denmark), and the PAR [16] index was measured by a single examiner previously calibrated. The Ortho Viewer program was used (3Shape, Copenhagen, Denmark).

The sample size calculation was performed using the G*Power software (Version 3.1.9.7–Düsseldorf, Germany), based on multiple linear regression, with an effect size of 0.2, bilateral alpha of 5%, a power of 80% and considering the maximum of four independent variables in the multiple linear regression model. The sample size totaled 65 patients.

### Statistical analysis

For method error analysis, the PAR index of 30 randomly selected initial models was calculated and reassessed after 15 days. Both casual and systematic errors were analyzed using the Bland–Altman method. The normality of residuals was assessed using the Q-Q residual plot. The univariate model was used to verify the relationship between the dependent variable (duration of orthodontic treatment) with the following variables: patient’s initial age, number of absences/missed appointments, number of debonds/breakages, number of missing teeth, sex, operator who performed treatment, the time in months that treatment was conducted during the pandemic and the time that the treatment was completed (before or during the pandemic). Those that presented a value of *p* < 0.20, in addition to the main independent variable (before/during the pandemic), were included in the multivariate model with the application of a multiple linear regression, which was complemented by the stepwise method. To compare the variables of patients treated before and during the pandemic, intergroup comparisons were performed using the independent t test and the Mann–Whitney test. Data normality was verified by the Shapiro–Wilk test. Data were evaluated using Jamovi software (version 2.3.9., Sydney, Australia).

## Results

### Participants

**Fig. 1:**
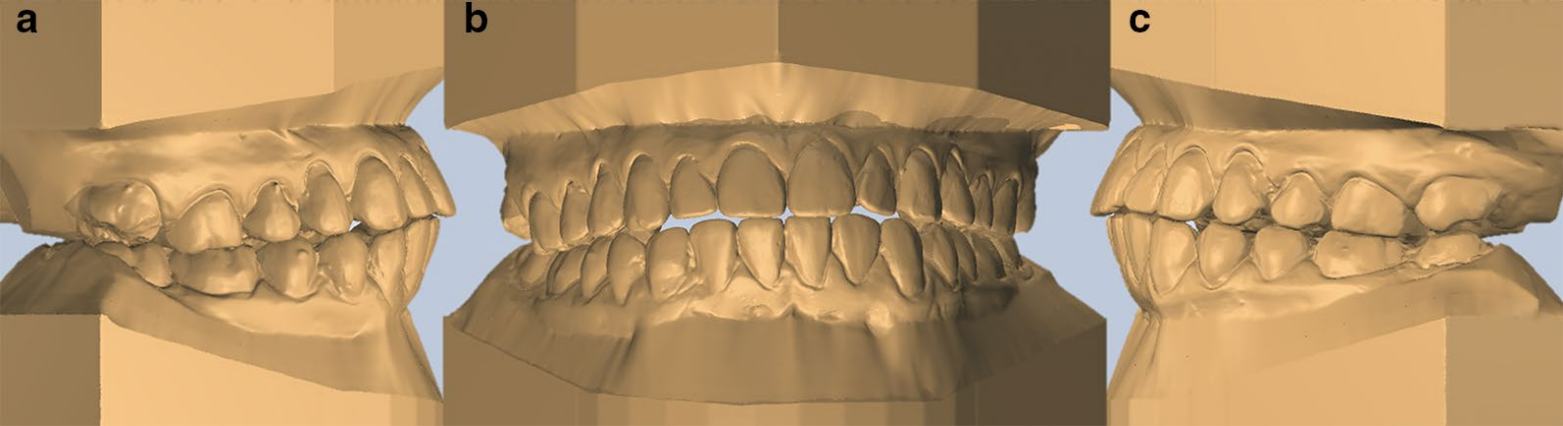
Case excluded due to poorly finalization. **a** Right view. **b** Frontal view. **c** Left view

**Fig. 2:**
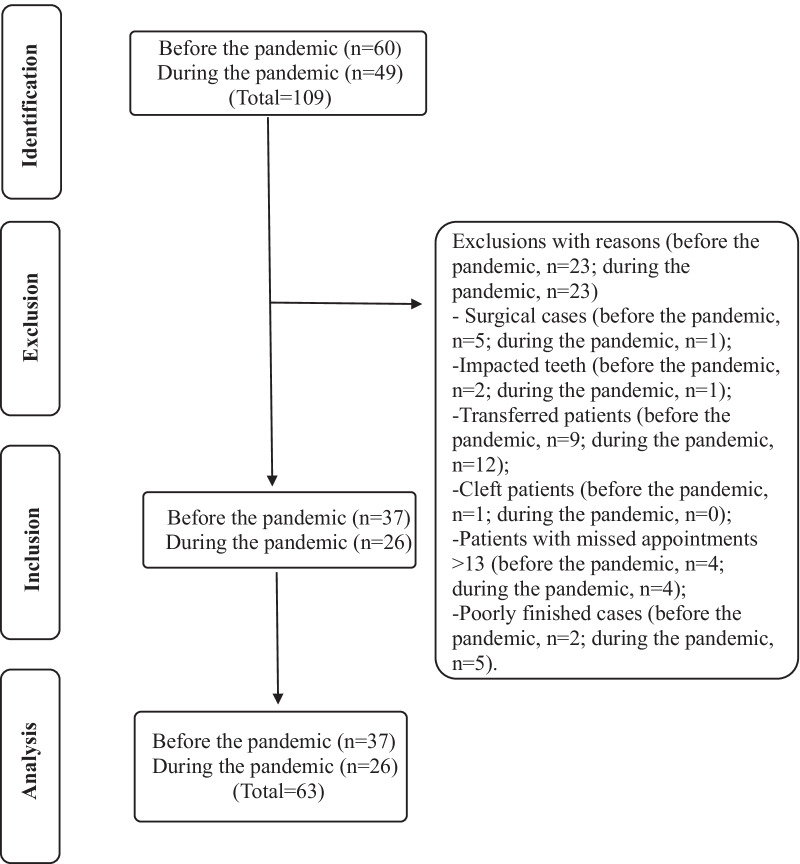
Flowchart of the selection of study participants

Initially, 109 patients’ records were evaluated, 60 from patients with treatment completed in the pre-pandemic period and 49 during the pandemic. The reasons for exclusions were: surgical cases (*n* = 6), cases with impacted teeth (*n* = 3), transferred patients (*n* = 21), cleft patients (*n* = 1), patients with > 13 missed appointments (*n* = 8) and poorly finished cases (*n* = 7) (Fig. 1). In the end, 63 patients were analyzed, 37 treated before, and 26 treated during the pandemic (Fig. 2).

### Descriptive data

Thirteen male patients (35.14%) and 24 female patients (64.86%) were included in the pre-pandemic group. Operator 1 presented 28 patients included in the sample, with a mean treatment time of 25.4 (± 9.6) months, and operator 2 presented 9 patients, with a mean treatment time of 36.8 (7.6) months. The mean treatment time including both operators’ patients was 28.2 (± 10.3) months. The initial age ranged from 10 to 59 years, with a median of 26.0 years, and the number of debonds/breakages ranged from 0 to 25 (median = 3). Five patients (13.5%) presented missing teeth. This variable ranged from 0 to 8 (median = zero), and the number of absences/missed appointments ranged from 0 to 11 (median = 11). Regarding the initial PAR index, the mean was 17.0 (± 6.0).

Regarding the patients that had the treatment completed during the pandemic, 12 male patients (46.15%) and 14 female patients (53.85%) were included. Operator 1 presented 14 patients included in the sample, with a mean treatment time of 26.4 (± 8.2) months, and operator 2 presented 12 patients, with a mean treatment time of 36.0 (± 9.3) months. The mean treatment time including both operators’ patients was 30.8 (± 9.8) months. The initial ages ranged from 12 to 56 years (median = 14), the number of debonds/breakages ranged from 0 to 17 (median = 3), and the number of absences/missed appointments ranged from 0 to 12 (median = 6). Three patients (11.5%) presented with missing teeth. This variable ranged between 0 and 7 (median = zero). The mean initial PAR index was 18.8 (± 6.1) (Table 1). The distribution of cases finished during the pandemic according to month and year is shown in Table 2.

**Table 1:** Descriptive analysis. Sample distribution, mean (SD), median (IQR) and minimum/maximum values for the variables

	Time that the treatment was finished	
	Before the pandemic	During the pandemic
Sample (n)	Operator 1 (n = 28) Operator 2 (n = 9) Total (n = 37)	Operator 1 (n = 14) Operator 2 (n = 12) Total (n = 26)
Treatment time Mean (SD)	Operator 1: 25.4 (9.6) Operator 2: 36.8 (7.6) Total: 28.2 (10.3)	Operator 1: 26.4 (8.2) Operator 2: 36.0 (9.3) Total: 30.8 (9.8)
Sex (M/F)	13 (35.14%) / 24 (64.86%)	12 (46.15%) / 14 (53.85%)
Patients with missing teeth	5 (13.5%)	3 (11.5%)
Missing teeth Median (IQR/Min–Max	0.0 (0.0)/0–8	0.0 (0.0)/0–7
Age *T*_0_ Median (IQR)/Min–Max	26.0 (25.0)/10–59	14.5 (14.0)/10–55
Debonds/breakages Median (IQR)/Min–Max	3.0 (6.0)/0–25	3.0 (8.5)/0–17
Absences/misses Median (IQR)/Min–Max	2.0 (3.0)/0–11	6.0(3.7)/0–12
PAR T_0_ Mean (SD)/Min/Max	17.0 (6.0)/4–31	18.8 (6.1)/6–30

*SD* Standard deviation and *DIQ* interquartile deviation

**Table 2:** Number of cases with treatment completed during the pandemic according to month and year

Month	Year		
	2020	2021	2022
January	–	1	0
February	–	1	1
March	0	2	0
April	0	0	2
May	0	2	–
June	1	1	–
July	0	1	–
August	2	1	–
September	3	1	–
October	1	0	–
November	4	0	–
December	0	2	–
Total	11	12	3

## Main results

**Fig. 3:**
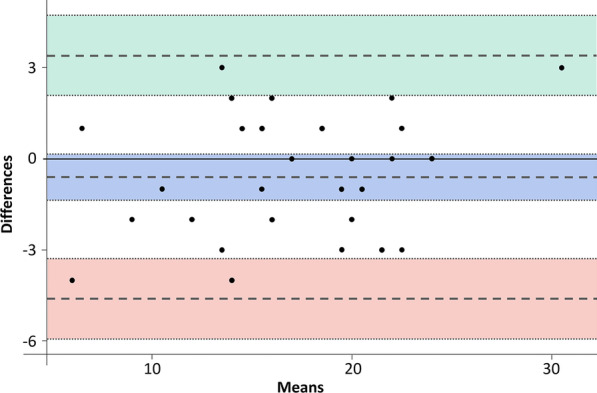
Bland–Altman scatter plot comparing PAR T0 index measurements performed at two different times

The error of the method was evaluated using the Bland–Altman plot by duplicating measurements of 30 pairs of models at T0. PAR index measurements at two different times were compared by the mean difference of the values obtained in each measurement. No significant systematic error (bias) was detected (bias = − 0.60. 95% confidence interval (CI) − 1.36 to 0.16). Precision (random error) ranged from − 4.60 (95%CI − 5.93 to − 3.28) to 3.40 (95%CI 2.09–4.72). Visually, there is a dispersion of the results within the CI, without the presence of outliers, illustrating a good agreement between the two measurements (Fig. 3).

**Fig. 4:**
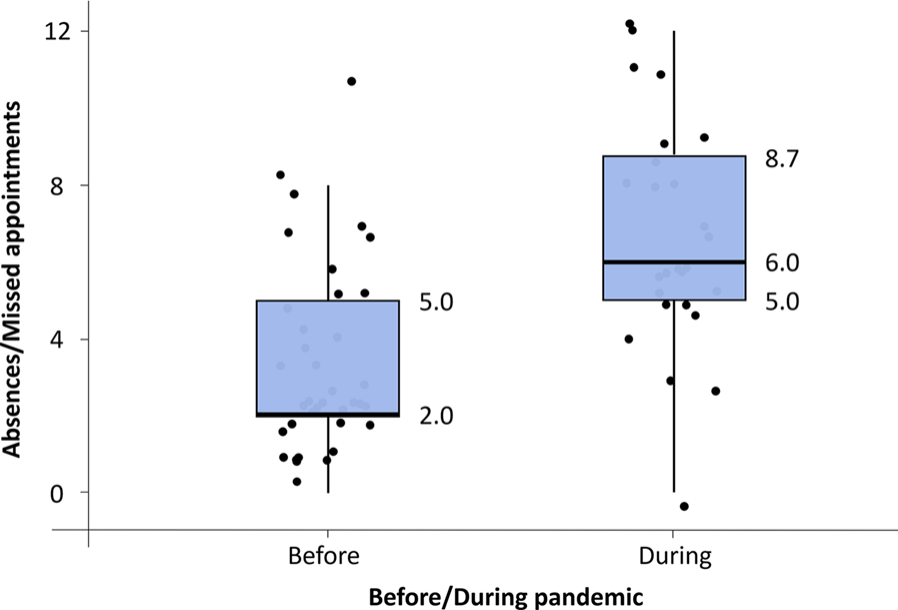
Box plot (median/DIQ) for absences/missed appointments of patients in the pre-pandemic and pandemic groups

Through the intergroup comparison, it was observed that the number of absences/missed appointments during the pandemic was significantly higher compared to those treated in the previous period (*p* < 0.001, Fig. 4, Table 3). The other variables showed no significant differences (Table 3).

**Table 3:** Comparison between patients treated before and during the pandemic through independent *t* test and Mann–Whitney test

Variable	Group	Mean/Median (SD/DIQ)	Difference	CI 95%		*p-*value
				Lower	Upper	
Absences/missed appointments	Before the pandemic	2.0 (3.0)	− 4.0	2.0	5.0	< 0.001*
	During the pandemic	6.0 (3.7)				
Age T_0_ (years)	Before the pandemic	26.0 (25.0)	11.5	− 13.0	1.0	0.2
	During the pandemic	14.5 (14.0)				
PAR T_0_	Before the pandemic	17.0 (6.0)	− 1.7	− 1.3	4.9	0.2
	During the pandemic	18.8 (6.1)				
Debonds/breakages	Before the pandemic	3.0 (6.0)	0.0	− 2.0	2.0	0.8
	During the pandemic	3.0 (8.5)				
Tooth loss	Before the pandemic	0.0 (0.0)	0.0	− 3.8	2.2	0.7
	During the pandemic	0.0 (0.0)				

*CI* 95% confidence interval, *SD* standard deviation, *DIQ* interquartile deviation

*Statistical significance *p* < 0.05

During the execution of the univariate analysis between the dependent variable, treatment time and the independent variables, a significant association was observed with the number of absences/missed appointments (*p* < 0.001), operator (*p* < 0.001) and gender (*p* = 0.04). Knowing that the number of absences/missed appointments had shown greater statistical significance through the Mann–Whitney comparison test in relation to when treatment was completed (before/during the pandemic), it was decided to remove the first variable from the multiple model in order to explore the second variable.

In the multiple linear regression model, gender showed a 4.7-month reduction in treatment time associated with females compared to males (*β* = 4.77, *p* = 0.03) and a 10-month reduction in the treatment time associated with operator 1 (*β* = 10.42, *p* = 0.03). Together, these two variables explained a 26% alteration in treatment time (adjusted *R*^2^ = 0.26, *p* < 0.001). The time that treatment was finished (before/during the pandemic) was included even though the significance level of *p* < 0.20 was not reached in the univariate model, as it is the independent variable of interest in this study. This variable had no impact on treatment time (*p* = 0.94). Stepwise regression showed a determination of 23% for the operator and 27% with the inclusion of sex (*p* < 0.001) (Table 4).

**Table 4:** Multiple linear regression model followed by the stepwise method

Independent variables	Univariate model	Multiple Model											
		Multiple								Stepwise			
	*p*-value	Adjusted *p-*value	CI 95%		Β value	*R*^2^	Adjusted *R*^2^	*F* test	*p-*value of multiple model	*p-*value of variables	*R*^2^	Adjusted*R*^2^	*p-*value of multiple model
			Lower	Upper									
Absences/missed appointments	< 0.001**	–	–	–	–					–	–	–	
Operator	< 0.001**	< 0.001**	5.64	15.22	10.42					< 0.001*	0.24	0.23	< 0.001**
Sex	0.04*	0.03*	0.25	9.29	4.77	0.30	0.26	8.43	< 0.001**	0.04*	0.30	0.27	< 0.001**
Treatment before/during the pandemic	0.31	0.94	− 4.76	4.45	− 0.15					0.94	0.30	0.26	< 0.001**
Months of treatment during the pandemic	0.63												
Missing teeth	0.67												
Age *T*_0_	0.81												
PAR *T*_0_	0.87												
Debonds/breakages	0.71												

*CI* 95% confidence interval

*Statistical significance *p* < 0.05, **p < 0.01

## Discussion

Some studies have already shown that orthodontics has been affected by the pandemic, especially regarding infection control measures and encouragement of virtual patient care [5, 17, 18]. In addition, the pandemic may have caused a reduction in the average number of new patients seeking orthodontic treatment in 2020 [19].

To fully understand the consequences of the new coronavirus in our specialty, the search for new knowledge not yet explored in this area is still necessary [20]. To date, only two studies have evaluated the impact of the pandemic on the duration of orthodontic treatment [12, 13], with divergent results. The first [12] found that despite the greater number of cancellations of patient appointments during the pandemic, this did not affect total treatment time, and this result is in line with what was found in this study. The second [13] found an increase of about four months in treatment time. None of these studies controlled confounding variables that might impact treatment time.

A previous study [7] showed that females were more anxious about the impact of the coronavirus on their orthodontic treatment than males, which may demonstrate that female individuals would be more zealous with their treatment. This could explain the shorter treatment time associated with females in the present study. This fact agrees with a previous study [21], but differs from others already published in which sex did not seem to be an important factor for treatment time [9–11].

The number of absences/missed appointments that occurred during the pandemic period was higher than that observed in the pre-pandemic period, which was already an expected fact. Therefore, even though the pandemic caused a greater number of patient absences/missed appointments, this did not seem to be enough to directly affect the total treatment time in a significant way. Through multiple linear regression, it was observed that even for the patients who received treatment for a greater number of months within the pandemic period, there was no impact on their total treatment time. This can demonstrate that although patients were more absent during this period due to restrictions and/or fear, they may have become more collaborative during their treatment. This is despite that they stayed at home for a long time or were using masks, facilitating their collaboration with the use of intermaxillary elastics, for example. This fact may have made their treatment times similar to those treated before the pandemic.

The use of remote monitoring for orthodontic treatment during the pandemic period has been discussed in the current literature [22–24]. This practice can make these patients feel more welcomed and, therefore, collaborative with their treatment. A systematic review carried out on this topic [14] found a study [24] that observed greater precision in tooth movement performed with orthodontic aligners when patients were monitored remotely. This same review noted that there are no controlled clinical trials using this tool in orthodontic treatment with fixed appliances, and further studies are needed on this topic. One of the orthodontists who contributed to the sample of this study (operator 1) used this device in some cases treated during the pandemic. This may also be a contributing factor to a non-significant increase in the treatment time of patients treated during this period. In addition, the experience and different habits of the two operators in their orthodontic routine, such as scheduling a smaller number of patients per day during the pandemic, may have contributed to more careful appointments and good management of the cases.

Several previous studies have shown that factors related to patient cooperation can directly impact their treatment time, including the number of absences [9–11, 21]. Knowing the great impact that this variable can have on treatment time, patients with more than 13 monthly absences/missed appointments were excluded from the sample to avoid possible outliers.

One study [21] demonstrated that debonds/breakages of orthodontic accessories caused by the patient seem to have less statistical significance in relation to the increase in treatment time than bracket repositioning performed as a decision of the orthodontist. The present study did not show significance in this association, probably because the median number of debonds/breakages of orthodontic accessories committed by these patients was minor. Previous data [25] demonstrated that orthodontic appliances may have failed more frequently during lockdown than normal times, demonstrating the greater variability of this variable according to the sample studied. It is also known that some orthodontists opt for passive reattachment of brackets after their debonding, thus avoiding the return to more flexible arches and possible delays in treatment. In addition, debonds/breakages of brackets or bands too close to patient appointment dates may not have a significant effect on treatment time.

Patients transferred from other professionals were excluded from the present sample. It has already been shown that this can be an important factor for increasing treatment time and poorer quality of finished cases [26]. This includes cases with impacted teeth, such as canines, which often need to be put in traction [27]. The impact of the operator on treatment time was clear in this study because of the greater statistical significance that the regression correction by this variable caused. It is imminent that different operators may cause different treatment times, even with similar clinical experiences, whether by different forms of planning or criteria for the finalization of cases.

The patients’ ages in the sample ranged from 10 to 59 years. A systematic review with a meta-analysis [28] showed that there is no difference in treatment time between adults and adolescents. This corroborates the findings of this study, which showed no effect of age on the variability of orthodontic treatment time. Histological differences between the adult and adolescent periodontium have been previously reported [29]. However, adults patients tend collaborate more with their consultations and adhere to the instructions of their orthodontist, counterbalancing biological factors that may interfere with the course of treatment.

In general, the present sample had few patients with missing teeth, despite this being a common clinical presentation, especially related to molar loss [30]. It has already been shown that patients with loss of permanent molars associated with space closure may have longer treatment times compared to patients without tooth loss [31]. In this study, this variable had no impact on treatment time. This can be explained not only by the low number of patients without missing teeth, but also by the choice of the orthodontist in most of the cases to keep the spaces for later prosthetic rehabilitation. The initial malocclusion was also not statistically significant in this study, corroborating other findings [9, 10, 31–34]. However, some studies have identified an influence of initial malocclusion on the treatment time variability [35–37], demonstrating that its prediction remains an inexact science and depends on numerous factors.

Previous studies [9, 31, 37] found no association in the quality of finished treatment with an increase or reduction of treatment time, while another two [38, 39] found a longer treatment time in cases with reduced clinical results. This can be justified by an “exhaustion of the patient” and consequent less collaboration or poor planning of the case. Cases considered poorly finished were excluded from this study to reduce the variability of the final PAR index and contributed to a greater homogeneity of the sample. These cases presented an index > 5, knowing that this value was according to the error margin found using the Bland–Altman method. The patients’ PAR index was calculated from the scans of the plaster models, and this method is considered valid and reliable [40]. Among seven excluded cases, five were mostly treated during the pandemic, thus indicating that a small portion of these patients may have anticipated their treatment completion.

## Limitations

This study presented a possible bias due to its retrospective nature. The cases of some of the patients, who had longer treatment times due to the pandemic, are still in progress. In addition, other factors not evaluated in this study may also contribute to the variability of treatment time, such as patient cooperation with the use of elastics.

## Conclusion

- The COVID-19 pandemic does not seem to have had a direct impact on the total time of orthodontic treatment; however, the number of absences/missed appointments was higher during this period.
- The variable that showed the greatest association with an increase in orthodontic treatment time was the orthodontist who conducted the treatment which explained 23% of the variability. The patient’s sex had a slight influence, increasing the determination to 27%.

## Data Availability

The datasets used and/or analyzed during the current study are available from the corresponding author on reasonable request.

## Abbreviations

CI: Confidence interval
DIQ: Interquartile deviation
IQR: Interquartile range
PAR: Peer Assessment Rating
SD: Standard deviation
WHO: World Health Organization
